# Differential expression of the epigenetic methylation-related protein DNMT1 by breast cancer molecular subtype and stromal histology

**DOI:** 10.1186/s12967-016-0840-x

**Published:** 2016-04-12

**Authors:** Eunah Shin, YuKyung Lee, Ja Seung Koo

**Affiliations:** Department of Pathology, CHA Gangnam Medical Center, CHA University, Seoul, South Korea; Department of Pathology, Yonsei University College of Medicine, 50 Yonsei-ro, Seodaemun-gu, Seoul, 120-752 South Korea

**Keywords:** Breast cancer, DNA methylation, DNMT1

## Abstract

**Background:**

We assessed the expression of methylation-related proteins 5-meC, DNMT1, and ISL-1 in breast cancer and evaluated their relationship to clinicopathological factors.

**Methods:**

Immunohistochemical staining for ER, PR, HER-2, Ki-67, 5-meC, DNMT1, and ISL-1 were performed on 348 breast cancer samples in tissue microarray. Samples were subgrouped into luminal A, luminal B, HER-2, or triple-negative breast cancer (TNBC) according to immunohistochemical staining for ER, PR, HER-2, and Ki-67. The tumor stroma was histologically subtyped into desmoplastic, sclerotic, normal-like, or inflammatory type.

**Results:**

Tumor expression of DNMT1 differed by molecular subtype: it was higher in TNBC and lower in luminal A (p < 0.001) samples. DNMT1 expression was also related to higher histologic grade, ER negativity, PR negativity, and higher Ki-67 LI (p < 0.001). In western blot, protein expressions of DNMT1 and ISL-1 were higher in TNBC and relatively lower in the remaining subtypes. High tumor expression of DNMT1 was associated with shorter OS in univariate analysis (p = 0.041). DNMT1 and 5-meC were differentially expressed by stromal phenotype: 5-meC was higher in normal-like type and lower in sclerotic type (p = 0.049); DNMT1 was higher in inflammatory and lower in sclerotic type (p < 0.001).

**Conclusions:**

Tumor expression of DNMT1 in breast cancer differed by molecular subtype and stromal histological type. DNMT1 was highly expressed in TNBC and in breast cancer with inflammatory stromal type.

## Background

One of the most important features differentiating cancer cells from normal cells is insensitivity to growth inhibitory signals. This insensitivity mostly occurs through the inhibition of tumor suppressor genes [1] by DNA hypermethylation. DNA hypermethylation is initiated by DNA methyltransferases (DNMTs) [2]. Genes for the DNMTs identified to date are *DNMT1*, *DNMT2*, *DNMT3A*, and *DNMT3B*. DNMT1 is a key maintenance methyltransferase and is the most common methyltransferase in humans. Molecules associated with DNMT1 are 5-methylcytosine (5-MeC) and insulin gene enhancer binding protein-1 (ISL-1). DNA methylation occurs when a methyl group is added to the 5′ position of the cytosine ring in CpG dinucleotides, yielding 5-MeC; thus, 5-MeC is the end product of DNA methylation. ISL-1 is a direct target of DNMT1 in breast cancer [3]. Previous studies of epigenetic methylation-related proteins in breast cancer found that expression of DNMT1 and DNMT3a is higher in breast cancer than in benign lesions and the expression is related to breast cancer prognosis [4]. However, breast cancers are heterogeneous tumors with diverse clinical, histological, and molecular features; thus, breast cancer is subgrouped into luminal A, luminal B, HER-2, normal breast-like, and basal-like types by gene profiling analysis [5, 6]. These molecular subgroups have different molecular, histological, and clinical features and differ in treatment response and prognosis. Breast cancer subtypes are expected to have differential expression of epigenetic methylation-related proteins, but studies on this topic have not yet been reported. We assessed the expression of methylation-related proteins 5-meC, DNMT1, and ISL-1 in breast cancer and evaluated their relationships with clinicopathological factors.

## Methods

### Patient selection and histological evaluation

Patients diagnosed with invasive ductal carcinoma, NOS, at Severance Hospital from January 2000 to December 2006 were included. Patients who received preoperative chemotherapy or hormonal therapy were excluded. This study was approved by the Institutional Review Board of Yonsei University Severance Hospital and was exempt from informed consent from patients. Hematoxylin and eosin (H&E)-stained slides of all cases were reviewed by a breast pathologist (Koo JS). Histological grade was assessed using the Nottingham grading system [7]. Clinicopathological parameters evaluated were patient age at initial diagnosis, lymph node metastasis, tumor recurrence, distant metastasis, and patient survival. Tumor stroma were subgrouped as: (1) desmoplastic type for tumor stroma with cellular fibroblast/myofibroblast proliferation; (2) sclerotic type for tumor stroma of fibrotic collagenous components with little cellular component; (3) normal-like type for stroma with no stromal reaction around tumor cells or for normal breast stroma; and (4) inflammatory type for tumor stroma composed of inflammatory cells such as lymphocytes.

### Tissue microarrays

A representative area showing the tumor and tumor stroma was selected on an H&E-stained slide and a corresponding spot was marked on the surface of the paraffin block. Using a biopsy needle, the selected area was punched out and a 3-mm tissue core was transferred to a 6 × 5 recipient block. Two tissue cores of invasive tumor were extracted to minimize extraction bias. Each tissue core was assigned a unique tissue microarray location number linked to a database containing clinicopathological data.

### Immunohistochemistry

Antibodies used for immunohistochemistry are listed in Table 1. Immunohistochemistry used formalin-fixed, paraffin-embedded 5-μm tissue sections obtained with a microtome, transferred onto adhesive slides, and dried at 62 °C for 30 min. After incubation with primary antibodies, immunodetection was performed with biotinylated anti-mouse immunoglobulin followed by peroxidase-labeled streptavidin using a labeled streptavidin–biotin kit with 3,3′-diaminobenzidine chromogen as the substrate. The primary antibody incubation step was omitted in the negative control. Positive control tissue was used as per the manufacturer’s recommendation. Slides were counterstained with Harris hematoxylin.

**Table 1:** Source, clone, and dilution of antibodies

Antibody	Company	Clone	Dilution
DNA methylation-related proteins			
DNMT1	Abcam, Cambridge, UK	2B5	1:200
5-meC	Abcam, Cambridge, UK	33D3	1:200
ISL-1	Abcam, Cambridge, UK	Polyclonal	1:200
Molecular subtype-related proteins			
ER	Thermo Scientific, San Diego, CA, USA	SP1	1:100
PR	DAKO, Glostrup, Denmark	PgR	1:50
HER-2	DAKO, Glostrup, Denmark	Polyclonal	1:1500
Ki-67	Abcam, Cambridge, UK	MIB	1:1000

### Interpretation of immunohistochemical staining

Immunohistochemical markers were assessed by light microscopy. A cut-off value of 1 % or more positively stained nuclei was used to define estrogen receptor (ER) and progesterone receptor (PR) positivity [8]. HER-2 staining was analyzed according to the American society of clinical oncology-college of American pathologists guidelines using the following categories: 0, no immunostaining; 1+, weak, incomplete membranous staining of less than 10 % tumor cells; 2+, complete membranous staining, either uniform or weak in at least 10 % of tumor cells; and 3+, uniform, intense membranous staining in at least 30 % of tumor cells [9]. HER-2 immunostaining was considered positive when strong (3+) membranous staining was observed; samples scored as 0 to 1+ were regarded as negative. Samples showing 2+ HER-2 expression were evaluated for HER-2 amplification by fluorescent in situ hybridization (FISH).

Immunohistochemical staining for 5-meC, DNMT1, and ISL1 was assessed semiquantitatively by light microscopy [10]. Staining results in malignant cells and stromal cells were assessed as 0, negative or weak immunostaining in <1 % of the tumor/stroma; 1, focal expression in 1–10 % of tumor/stroma; 2, positive in 11–50 % of tumor/stroma; and 3, positive in 51–100 % of tumor/stroma. This evaluation was applied to all areas of the tumor in all samples; grade 0 was negative and grades higher than 1 were positive. Positive results were further classified as low (grades 1 and 2) and high (grade 3).

### Tumor phenotype classification

We classified breast cancer phenotypes according to immunohistochemical results for ER, PR, HER-2, and Ki-67 and FISH results for HER-2 [11]: luminal A type, ER or/and PR positive, HER-2 negative, and Ki-67 LI <14 %; luminal B type (HER-2 negative), ER or/and PR positive, HER-2 negative and Ki-67 LI ≥14 %; luminal B type (HER-2 positive), ER or/and PR positive and HER-2 overexpressed or/and amplified; HER-2 overexpression type, ER and PR negative and HER-2 overexpressed or/and amplified; and TNBC type, ER, PR, and HER-2 negative.

#### Laser microdissection, protein extraction from FFPE tissues and Western blot

To acquire tumor, laser microdissection was performed with hematoxylin stained uncovered slides generated by FFPE blocks (LMD 6500, Leica, Wetzlar,Germany). Five cases per each molecular subtype of breast cancer were miscrodissected. Protein extractions from formalin-fixed, paraffin-embedded (FFPE) tissues were performed using the Qproteome FFPE tissue kit (Qiagen, Hilden, Germany). Briefly, three sections from the same block were deparaffinized in xylene and rehydrated in graded series of alcohol. The tissues were mixed with FFPE extraction buffer (EXB), incubated at 100 °C for 20 min and at 80 °C for 2 h with agitation at 750 rpm, and then centrifuged for 15 min at 14,000×*g* at 4 °C. The supernatant containing the extracted proteins were determined by the Bradford assay (Bio-Rad Laboratories, Hercules, CA). An equal amount of protein from each sample extract was separated on SDS-PAGE gels and blotted onto nitrocellulose membranes (Bio-Rad). Western blotting was performed with primary antibodies against Dnmt 1, Islet 1, and actin (Abcam, Cambridge, UK), and specific bands were detected using the enhanced chemiluminescence kit (GE Healthcare Life Sciences, Little Chalfont, UK).

### Statistical analysis

Data were analyzed using SPSS for Windows, Version 12.0 (SPSS Inc., Chicago, IL, USA). For determination of statistical significance, Student’s t test and Fisher’s exact test were used for continuous and categorical variables, respectively. To analyze data with multiple comparisons, a corrected p-value with application of the Bonferroni multiple comparison procedure was used. Statistical significance was set at p < 0.05. Kaplan–Meier survival curves and log-rank statistics were used to evaluate time to tumor recurrence and overall survival. Multivariate regression analysis used the Cox proportional hazards model.

## Results

### Basal characteristics of breast cancer

Among the 348 breast cancer samples in this study, 162 (42.8 %) were luminal A, 84 (23.7 %) were luminal B, 27 (9.0 %) were HER-2 type, and 75 (24.5 %) were TNBC. Upon evaluation of clinicopathologic parameters, histologic grade, KI-67 LI, and stromal phenotype were different according to the molecular subtype with statistical significance (p < 0.001). TNBC was associated with higher histological grade and higher Ki-67 LI than other subtypes (Table 2). Luminal B demonstrated a higher percentage of desmoplastic stromal type than other subtypes, whereas TNBC demonstrated a higher percentage of inflammatory type.

**Table 2:** Clinicopathological characteristics of patients by breast cancer molecular subtype

Parameter	Total (n = 348) (%)	Luminal A (n = 162) (%)	Luminal B (n = 84) (%)	HER-2 (n = 27) (%)	TNBC (n = 75) (%)	*P* value
Age (years)						
≤50	202 (58.0)	94 (58.0)	55 (65.5)	13 (48.1)	40 (53.3)	0.299
>50	146 (46.7)	68 (42.0)	29 (34.5)	14 (51.9)	35 (46.7)	
Histological grade						
I/II	242 (69.5)	147 (90.7)	53 (63.1)	12 (44.4)	30 (40.0)	<*0.001*
III	106 (30.5)	15 (9.3)	31 (36.9)	15 (55.6)	45 (60.0)	
Tumor stage						
T1	182 (52.3)	96 (59.3)	42 (50.0)	13 (48.1)	31 (41.3)	0.068
T2/T3	166 (47.7)	66 (40.7)	42 (50.0)	14 (51.9)	44 (58.7)	
Nodal metastasis						
Absent	208 (59.8)	94 (58.0)	48 (57.1)	17 (63.0)	49 (65.3)	0.676
Present	140 (40.2)	68 (42.0)	36 (42.9)	10 (37.0)	26 (34.7)	
Estrogen-receptor status						
Negative	107 (30.7)	2 (1.2)	3 (3.6)	27 (100.0)	75 (100.0)	<*0.001*
Positive	241 (69.3)	160 (98.8)	81 (96.4)	0 (0.0)	0 (0.0)	
Progesterone-receptor status						
Negative	149 (42.8)	20 (12.3)	27 (32.1)	27 (100.0)	75 (100.0)	<*0.001*
Positive	199 (57.2)	142 (87.7)	57 (67.9)	0 (0.0)	0 (0.0)	
HER-2 status						
Negative	280 (80.5)	162 (100.0)	43 (51.2)	0 (0.0)	75 (100.0)	<*0.001*
Positive	68 (19.5)	0 (0.0)	41 (48.8)	27 (100.0)	0 (0.0)	
Ki-67 LI (%)						
≤14	213 (61.2)	162 (100.0)	24 (28.6)	13 (48.1)	14 (18.7)	<*0.001*
>14	135 (38.8)	0 (0.0)	60 (71.4)	14 (51.9)	61 (81.3)	
Stromal phenotype						
Desmoplastic type	97 (27.9)	42 (25.9)	34 (40.5)	6 (22.2)	15 (20.0)	<*0.001*
Inflammatory type	30 (8.6)	3 (1.9)	4 (4.8)	3 (11.1)	20 (26.7)	
Normal-like type	16 (4.6)	7 (4.3)	4 (4.8)	0 (0.0)	5 (6.7)	
Sclerotic type	205 (58.9)	110 (67.9)	42 (50.0)	18 (66.7)	35 (46.7)	
Tumor recurrence	32 (9.2)	10 (6.2)	8 (9.5)	3 (11.1)	11 (14.7)	0.204
No. of patient deaths	35 (10.1)	9 (5.6)	9 (10.7)	4 (14.8)	13 (17.3)	*0.033*
Duration of clinical follow-up (months, mean ± SD)	77.0 ± 33.8	78.4 ± 31.9	75.2 ± 33.5	77.7 ± 39.5	75.7 ± 36.3	0.884

*TNBC* triple negative breast cancer

### Expression of epigenetic methylation-related proteins in breast cancer

**Fig. 1:**
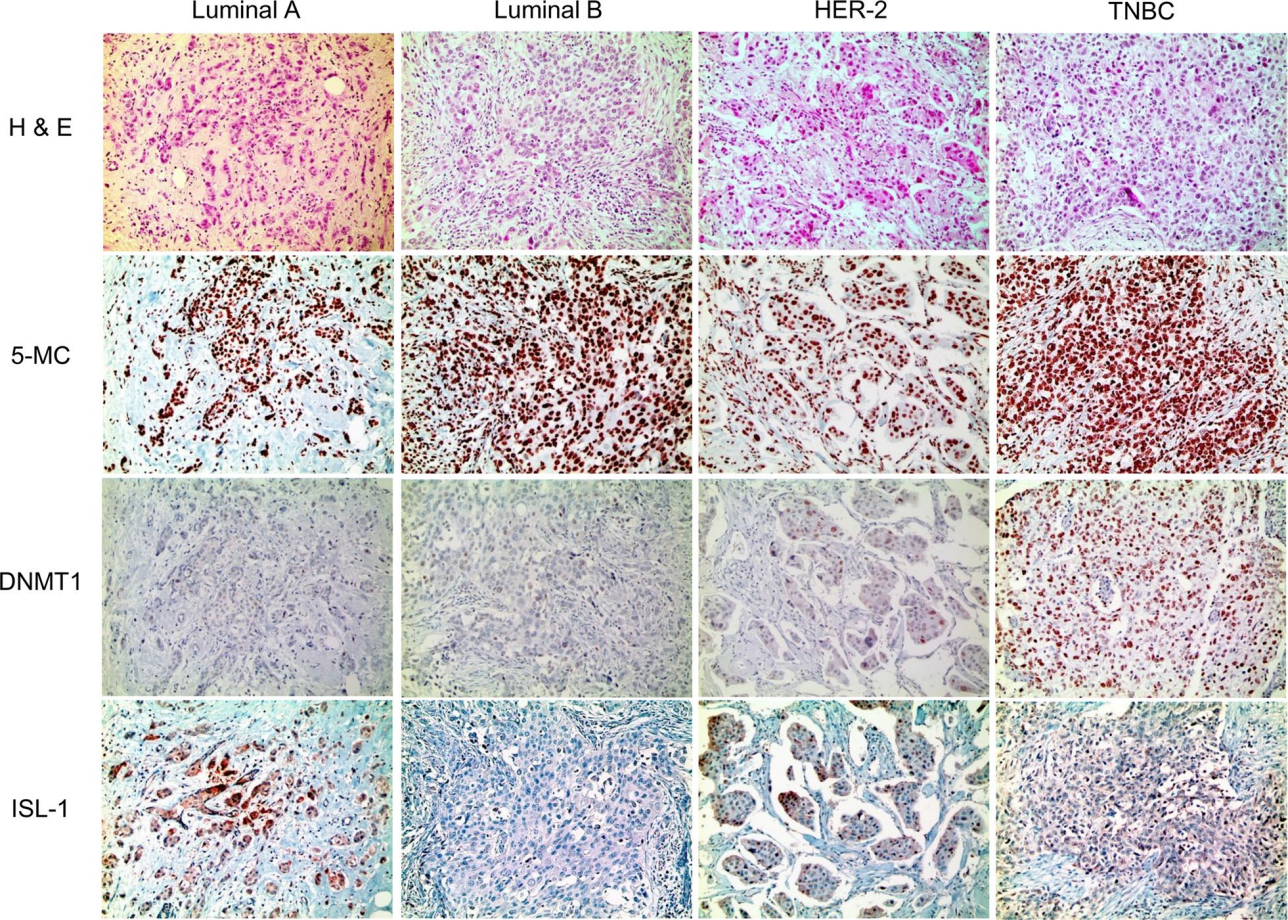
Expression of epigenetic methylation-related proteins by breast cancer molecular subtype. Expression of DNMT1 in malignant cells is higher in TNBC and lower in luminal A. Expression of DNMT1 in stromal cells is apparent in TNBC only

**Fig. 2:**
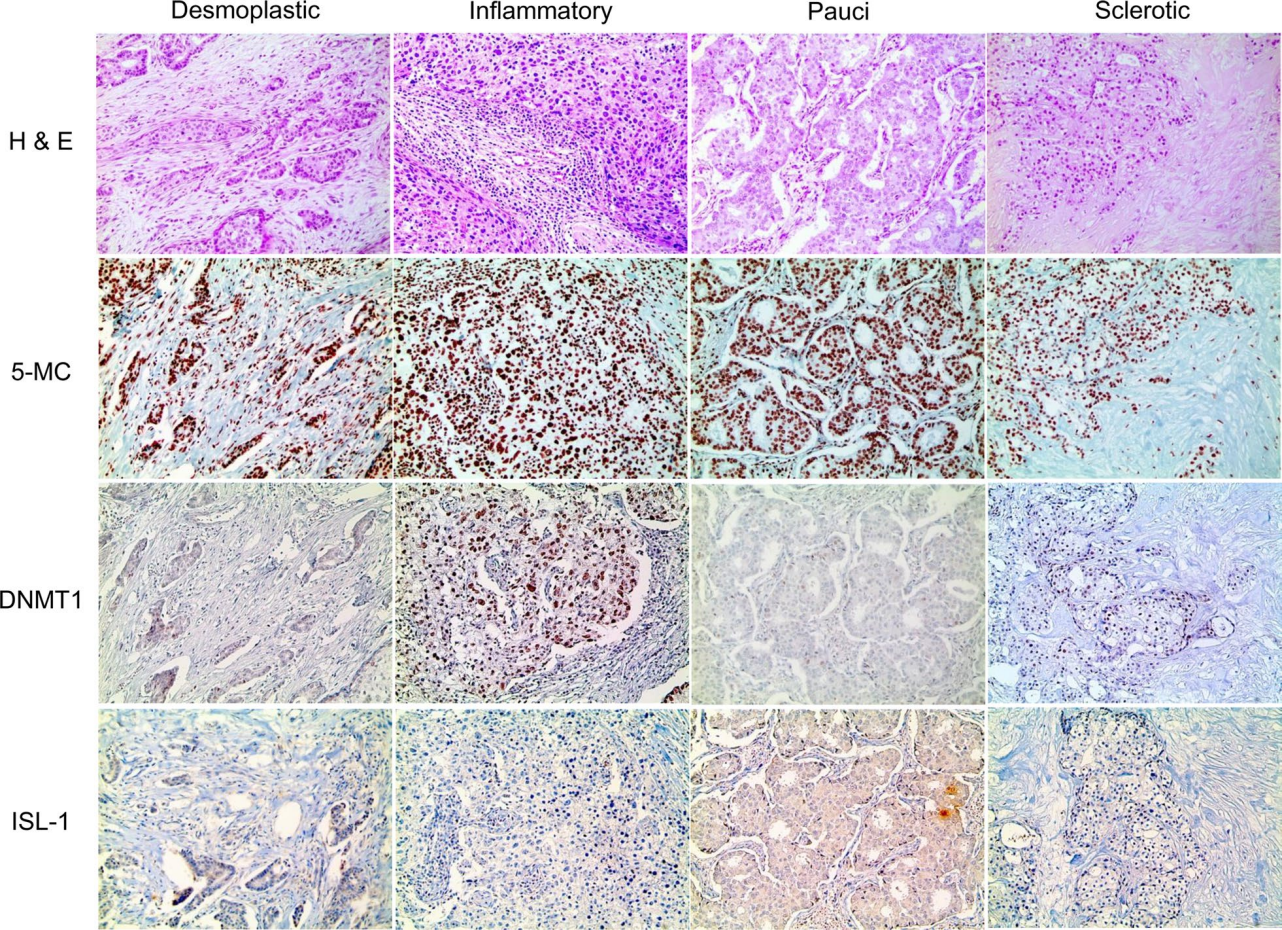
Expression of epigenetic methylation-related proteins by breast cancer stromal phenotype. 5-meC expression in malignant cells is highly expressed in normal-like type, with lower expression in sclerotic type. In addition, expression of DNMT1 in malignant cells is higher in inflammatory type and lower in sclerotic type

**Fig. 3:**
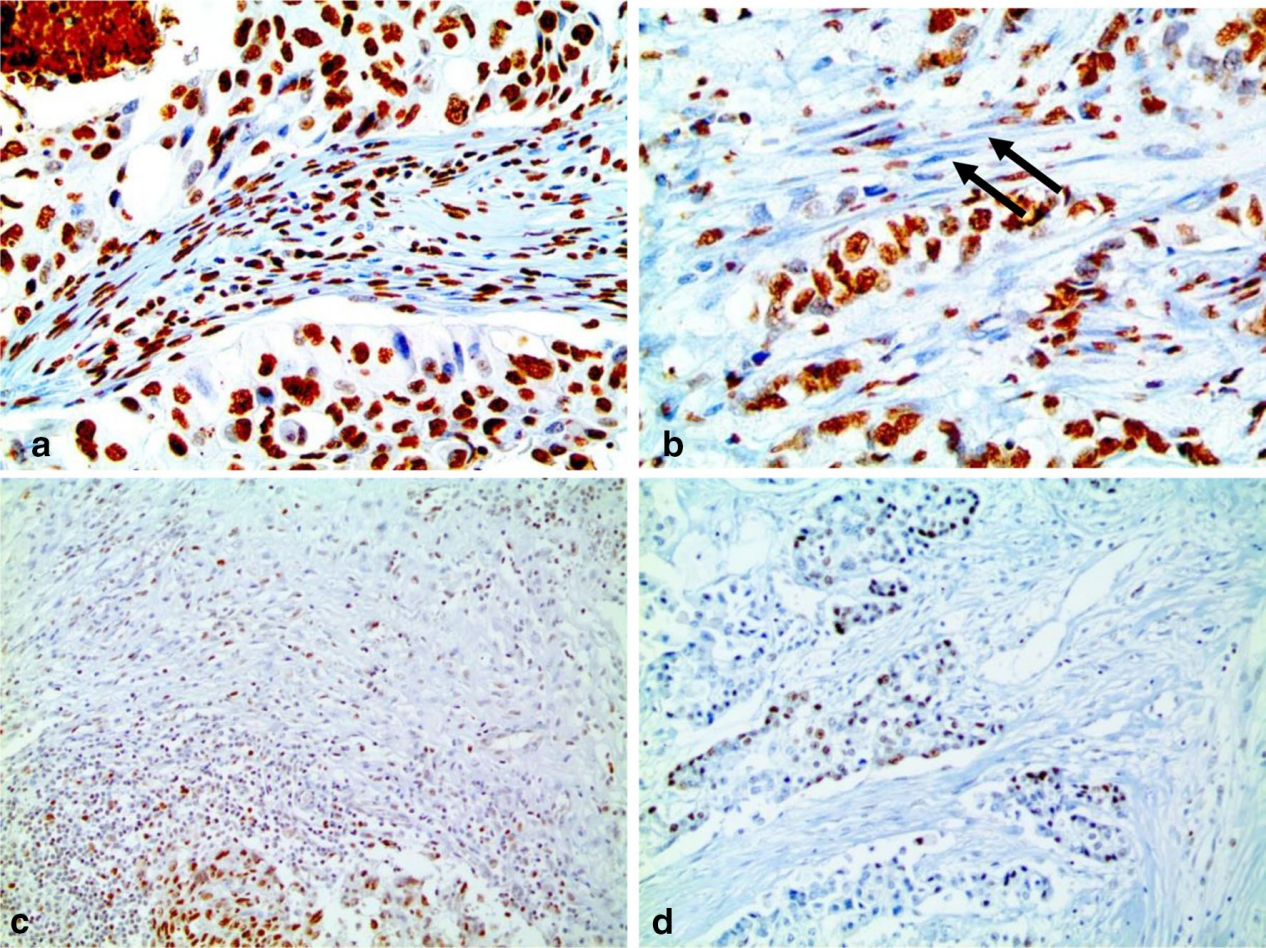
Representative images for 5-meC (**a**, **b**) and DNMT1 (**c**, **d**) in breast cancer stroma (**a**) tumor cells and tumor stromal cells showing a positive reaction to 5-meC, **b** tumor cells positive for 5-meC, but with spindle cells (*arrow*) negative to 5-meC within the stroma. **c** Tumor cells and tumor stromal cells were positive for DNMT1; **d** tumor cells positive to DNMT1, but with negative stromal cells

The epigenetic methylation-related proteins 5-meC and DNMT1 were expressed in both malignant cells and stromal cells, and ISL-1 was expressed only in the malignant cells. Expression analysis of epigenetic methylation-related proteins according to the molecular subtypes revealed that the expression of DNMT1 in malignant cells differs by molecular subtype (p < 0.001): it was higher in TNBC and lower in luminal A (Table 3 and Fig. 1). Expression of DNMT1 in stromal cells was apparent in TNBC only. Meanwhile, expression analysis of epigenetic methylation-related proteins according to the stromal phenotypes demonstrated that the expressions of 5-meC and DNMT1 in malignant cells differ by stromal phenotype (p = 0.049, and p < 0.001, respectively): 5-meC expression in malignant cells was highly expressed in normal-like type, with lower expression in sclerotic type. Expression of DNMT1 in malignant cells was higher in inflammatory type and lower in sclerotic type (Table 4 and Fig. 2). Fourteen cases (4 %) disclosed low positive results for 5-meC in the stromal component, in which spindle cells with a negative reaction to 5-meC were seen (Fig. 3). Only two cases (0.6 %) had a positive reaction to DNMT1 in the stromal cells.

**Table 3:** Expression of epigenetic methylation-related proteins by breast cancer subtype

Parameter	Total (n = 348) (%)	Luminal A (n = 162) (%)	Luminal B (n = 84) (%)	HER-2 (n = 27) (%)	TNBC (n = 75) (%)	*P* value
5-meC (T)						
Low	44 (12.6)	27 (16.7)	8 (9.5)	3 (11.1)	6 (8.0)	0.200
High	304 (87.4)	135 (83.3)	76 (90.5)	24 (88.9)	69 (92.0)	
5-meC (S)						
Low	14 (4.0)	7 (4.3)	2 (2.4)	1 (3.7)	4 (5.3)	0.810
High	334 (96.0)	155 (95.7)	82 (97.6)	26 (96.3)	71 (94.7)	
DNMT1 (T)						
Low	316 (90.8)	161 (99.4)	77 (91.7)	24 (88.9)	54 (72.0)	<*0.001*
High	32 (9.2)	1 (0.6)	7 (8.3)	3 (11.1)	21 (28.0)	
DNMT1 (S)						
Negative	346 (99.4)	162 (100.0)	84 (100.0)	27 (100.0)	73 (97.3)	0.062
Positive	2 (0.6)	0 (0.0)	0 (0.0)	0 (0.0)	2 (2.7)	
ISL-1 (T)						
Negative	343 (98.6)	159 (98.1)	84 (100.0)	27 (100.0)	73 (97.3)	0.455
Positive	5 (1.4)	3 (1.9)	0 (0.0)	0 (0.0)	2 (2.7)

**Table 4:** Expression of epigenetic methylation-related proteins by stromal phenotype

Parameter	Total (n = 348) (%)	Desmoplastic type (n = 97) (%)	Inflammatory type (n = 30) (%)	Normal-like type (n = 16) (%)	Sclerotic type (n = 205) (%)	*P* value
5-meC (T)						
Low	44 (12.6)	8 (8.2)	2 (6.7)	0 (0.0)	34 (16.6)	*0.049*
High	304 (87.4)	89 (91.8)	28 (93.3)	16 (100.0)	171 (83.4)	
5-meC (S)						
Low	14 (4.0)	3 (3.1)	1 (3.3)	0 (0.0)	10 (4.9)	0.726
High	334 (96.0)	94 (96.9)	29 (96.7)	16 (100.0)	195 (95.1)	
DNMT1 (T)						
Low	316 (90.8)	88 (90.7)	21 (70.0)	14 (87.5)	193 (94.1)	<*0.001*
High	32 (9.2)	9 (9.3)	9 (30.0)	2 (12.5)	12 (5.9)	
DNMT1 (S)						
Negative	346 (99.4)	97 (100.0)	29 (96.7)	16 (100.0)	204 (99.5)	0.197
Positive	2 (0.6)	0 (0.0)	1 (3.3)	0 (0.0)	1 (0.5)	
ISL-1 (T)						
Negative	343 (98.6)	96 (99.0)	29 (96.7)	16 (100.0)	202 (98.5)	0.775
Positive	5 (1.4)	1 (1.0)	1 (3.3)	0 (0.0)	3 (1.5)

### Differences in clinicopathologic factors according to the expression status of epigenetic methylation-related proteins

**Fig. 4:**
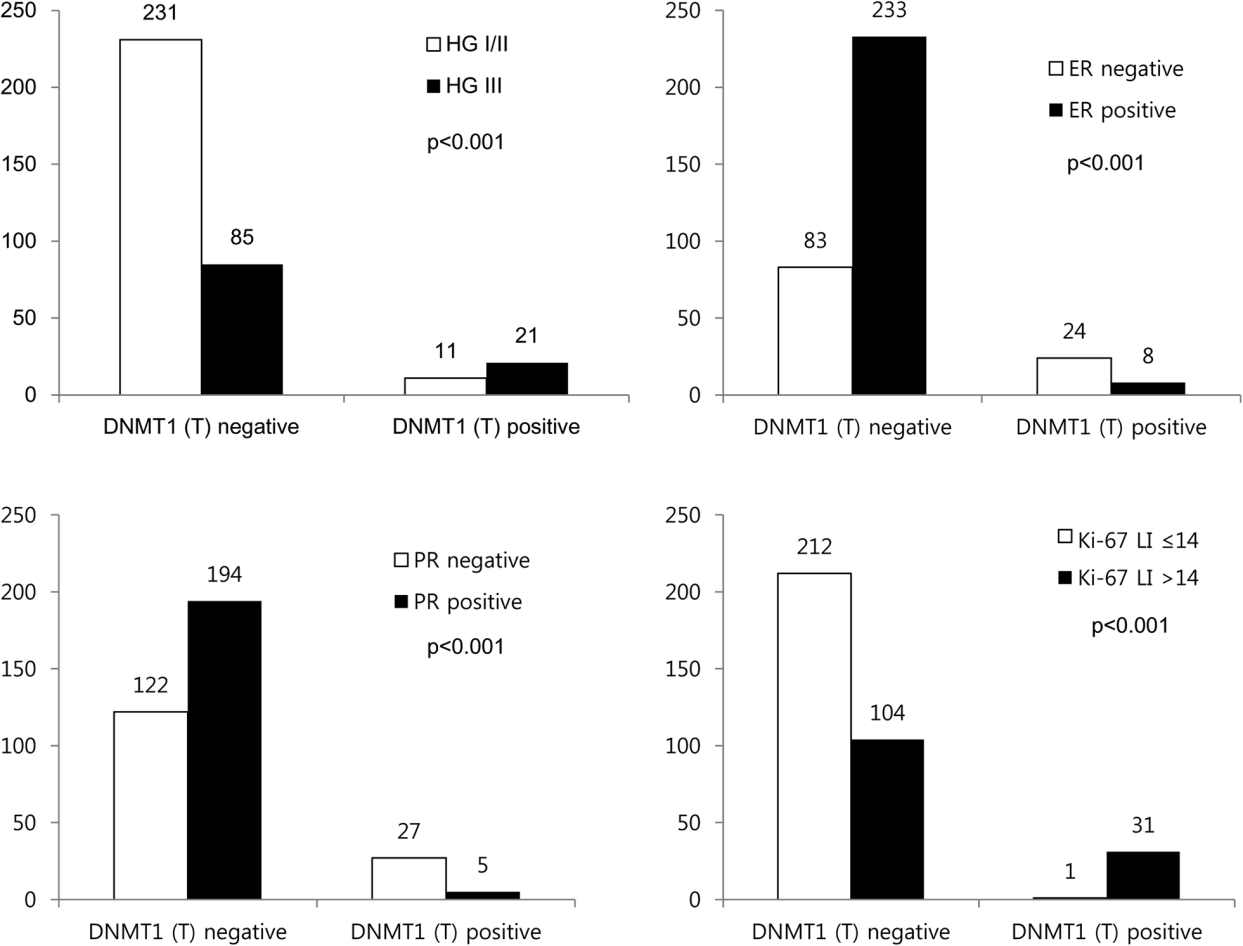
Differences of clinicopathological factors according to the expression of DNMT1 in malignant cells. Expression of DNMT1 in malignant cells is associated with higher histological grade, ER negativity, PR negativity, and higher Ki-67 LI

When clinicopathologic parameters were evaluated according to the expression status of epigenetic methylation-related proteins, expression of DNMT1 in malignant cells was associated with higher histological grade, ER negativity, PR negativity, and higher Ki-67 LI (p < 0.001) (Fig. 4).

### Impact of epigenetic methylation-related protein expression in breast cancer on patient prognosis

**Fig. 5:**
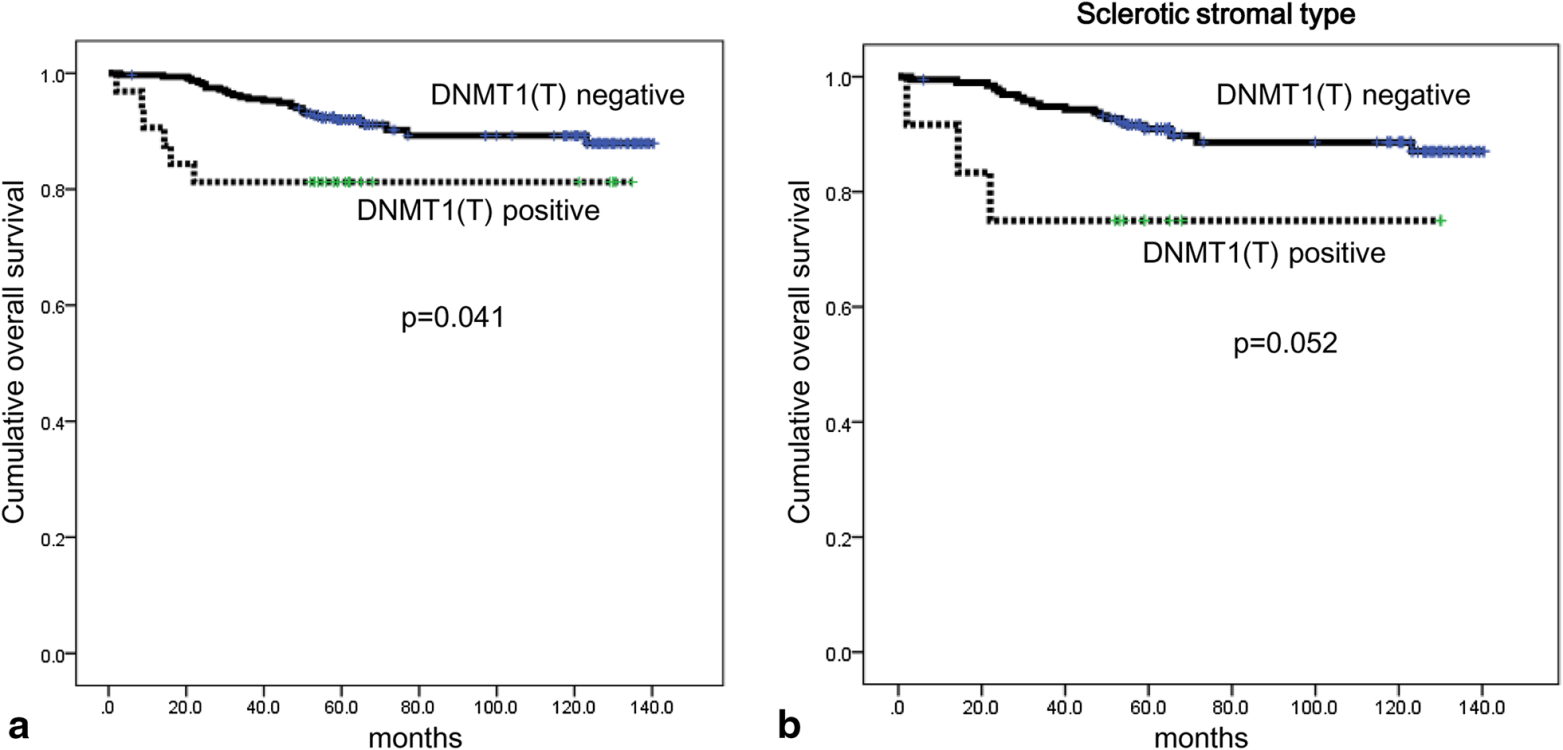
Overall survival for breast cancer (**a**) and breast cancer with sclerotic stromal type (**b**) according to DNMT1 status in malignant cells

Univariate analysis of the impact of epigenetic methylation-related protein expression on breast cancer patient prognosis showed that high expression of DNMT1 in malignant cells correlated with shorter OS (p = 0.041) (Table 5 and Fig. 5a); however, the association was not significant in multivariate Cox analysis (Table 6). Univariate analysis of the impact of epigenetic methylation-related protein expression by stromal phenotype on patient prognosis showed that high expression of DNMT1in malignant cells tended to be associated with shorter OS in sclerotic type (p = 0.052, Fig. 5b).

**Table 5:** Univariate analysis of the impact of epigenetic methylation-related protein expression on breast cancer patient prognosis by log-rank test

Parameter	Number of patients/recurrence/death	Disease-free survival		Overall survival	
		Mean survival (95 % CI) months	*P* value	Mean survival (95 % CI) months	*P* value
5-meC (T)					
Low	44/2/3	131 (124–138)	0.206	132 (124–141)	0.370
High	304/30/32	126 (121–130)		127 (123–132)	
5-meC (S)					
Low	14/1/1	129 (115–143)	0.709	131 (116–145)	0.659
High	334/31/34	127 (122–131)		128 (124–132)	
DNMT1 (T)					
Low	316/28/29	127 (123–131)	0.348	129 (126–133)	*0.041*
High	23/4/6	115 (100–129)		111 (95–128)	
DNMT1 (S)					
Negative	346/32/35	N/A	N/A	N/A	N/A
Positive	2/0/0	N/A		N/A	
ISL-1 (T)					
Negative	343/32/34	N/A	N/A	128 (124–132)	0.436
Positive	5/0/1	N/A		63 (60–66)

**Table 6:** Multivariate analysis of breast cancer survival

Included parameters	Disease-free survival			Overall survival		
	Hazard ratio	95 % CI	*P* value	Hazard ratio	95 % CI	*P* value
T stage						
T1 versus T2–3	2.376	1.064–5.305	*0.035*	1.714	0.826–3.556	0.148
N stage						
N0 versus N1–3	2.994	1.424–6.296	*0.004*	2.127	1.061–4.262	*0.033*
Histologic grade						
I/II versus III	1.214	0.515–2.859	0.658	0.785	0.353–1.747	0.553
ER status						
Negative versus positive	0.544	0.197–1.507	0.242	0.650	0.259–1.632	0.359
PR status						
Negative versus positive	0.768	0.275–2.147	0.615	0.436	0.164–1.158	0.096
HER-2 status						
Negative versus positive	0.967	0.406–2.307	0.940	1.050	0.463–2.381	0.907
Ki-67 LI						
≤14 versus >14	0.764	0.308–1.899	0.563	0.805	0.340–1.901	0.620
5-meC (T)						
Low versus high	3.117	0.728–13.35	0.126	1.828	0.543–6.155	*0.330*
5-meC (S)						
Low versus high	1.498	0.201–11.13	0.693	1.515	0.203–11.31	*0.686*
DNMT1 (T)						
Low versus high	0.937	0.289–3.037	0.913	1.473	0.538–4.036	0.451
DNMT1 (S)						
Negative versus positive	N/A	N/A	N/A	N/A	N/A	N/A
ISL-1 (T)						
Negative versus positive	N/A	N/A	N/A	2.075	0.270–15.96	0.483

#### Western blot analysis of the epigenetic methylation-related protein in breast cancer according to the molecular subtype

**Fig. 6:**
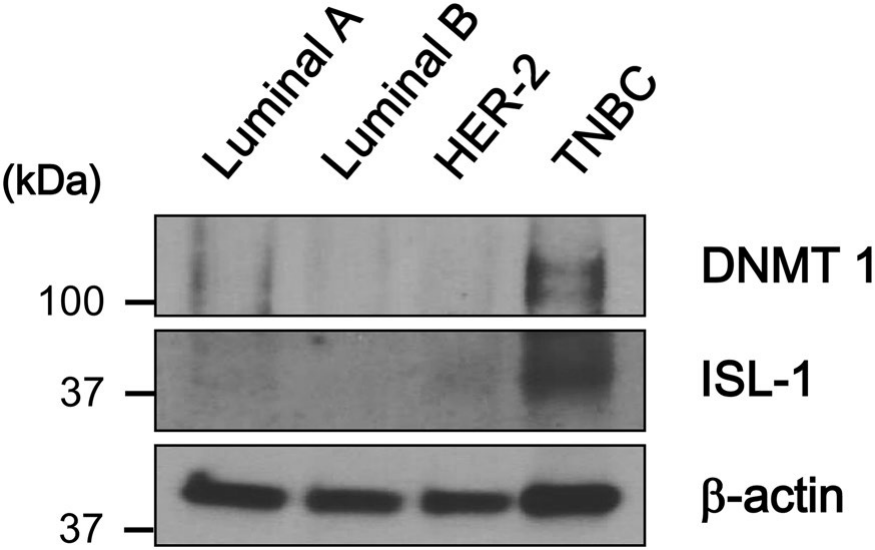
Western blot analysis of DNMT1 and ISL-1 in *FFPE breast cancer tissue* according to the molecular subtype. Protein expressions of DNMT1 and ISL-1 are higher in TNBC and relatively lower in the remaining subtypes

Western blot analysis was performed to confirm the expression of epigenetic methylation-related protein according to the breast cancer molecular subtypes. Protein expressions of DNMT1 and ISL-1 were higher in TNBC and relatively lower in the remaining subtypes (Fig. 6).

## Discussion

We assessed the expression status of epigenetic methylation-related proteins in breast cancer. Tumor expression of DNMT1 differed with breast cancer molecular subtype. Our results identified high tumor expression of DNMT1 in TNBC and low tumor expression of DNMT 1 in luminal A type, concordant with a previous study in which DNMT1 expression was reported to be increased in breast cancer compared to other benign lesions, especially in ER-negative breast cancer [4]. DNMT1 expression in our study was also correlated with ER negativity and PR negativity. A possible mechanism of higher expression of DNMT1 in TNBC might be association of TNBC with cancer stem cells. TNBC is highly correlated with cancer stem cell characteristics and DNMT1 is essential for cancer cell maintenance, resulting in high expression of DNMT1 in TNBC [3]. Our results showed that the expression of epigenetic methylation-related proteins differ by stromal phenotype. Tumor expression of DNMT1 was higher in inflammatory stromal type samples compared to other subtypes. Previous studies reported that IL-6 increases nuclear translocation of DNMT1 through phosphorylation of the nuclear localization sequence [12]. IL-6 is mainly secreted by tumor-infiltrating lymphocytes [13] and tumor-associated macrophages [14], explaining the increased expression of DNMT1 in the inflammatory stromal subtype. These results imply that stromal subtype might affect the methylation status of breast cancer. However, further study is needed to test this hypothesis.

DNMT1 and 5-meC were expressed in both malignant cells and stromal cells, although with different expression status; 5-meC was expressed in the stromal cells in 96 %, whereas DNMT1 was expressed in only less than 1 %. An important factor in the tumor microenvironment is cancer-associated fibroblasts (CAFs). Epigenetic alteration can occur in CAFs, as seen by differences in specific DNA methylation patterns between tumor-associated stroma and non-tumor stroma on methylation pattern analysis [15, 16]. About 4 % of the cases in our study had spindle-shaped stromal cells that were non-immunoreactive to 5-meC. These 5-meC-negative spindle cells are presumed to be hypomethylated CAFs, but this possibility needs to be tested with further study. The expression of DNMT1 was mostly negative in the tumor stroma, compatible with previous results showing no or low immunohistochemical reaction to DNMT1 in the tumor stroma and normal stroma [17].

We found an association between high tumor expression of DNMT1 and shorter OS, concordant with previous studies in which high expression of DNMT1 was associated with poor prognosis in malignant lymphoma [10], renal cell carcinoma [18], pancreatic cancer [19], and bladder cancer [20]. The clinical significance of this result is the possibility of therapy targeting epigenetic methylation-related proteins such as DNMT1. Currently, the possibility of DNMT1 as a new target is being explored for many tumors [21–24] and DNMT1 can be a new target for breast cancer as well. Since the expression degree of DNMT1 differs by molecular and stromal subtypes, DNMT1 may very well be a new therapeutic target for breast cancer type with high DNMT1 expression.

## Conclusion

In conclusion, DNMT1 is differentially expressed in breast cancer according to the molecular and stromal subtypes, and it is most highly expressed in TNBC and inflammatory stromal types.

## Abbreviations

DNMT: DNA methyltransferase
5-MeC: 5-methylcytosine
ISL-1: insulin gene enhancer binding protein-1, 2
H&E: hematoxylin and eosin
ER: estrogen receptor
PR: progesterone receptor
